# The anti-aging potential of VA’S new derivatives through metabolomic profiling

**DOI:** 10.1038/s41598-025-25545-9

**Published:** 2025-11-27

**Authors:** Li Tan, Jiang Wu, Jiewen Wang, Hongkang Zhang, Wei Zhang, Ang Gao, Zhijian Zhong, Tie-gen Chen, Baoping Zhou, Tengfei Han, Die Zhang, Miao He, Zhenxin Fan, Ya Zhou

**Affiliations:** 1 The Conservation of Endangered Wildlife Key Laboratory of Sichuan Province, College of Life Sciences, Sichuan University, Chengdu, China; 2 Shanghai Coachchem Technology, Shanghai, China; 3 Institute of Blood Transfusion, Chinese Academy of Medical Sciences, Chengdu, Sichuan China; 4 Shanghai Institute of Materia Medica, Chinese Academy of Sciences, Shanghai, China

**Keywords:** MVA, Metabolomics, Anti-aging, Molecular synthesis, Senescence, Metabolomics

## Abstract

Skin aging is a complex process influenced by intrinsic and extrinsic factors, leading to structural and functional changes such as reduced elasticity and increased wrinkles. This study aims to evaluate the potential biological functions of Magic vitamin A (MVA) in skin health management and skin aging through metabolomics analysis. We performed untargeted metabolomics analysis using liquid chromatography-tandem mass spectrometry (LC–MS/MS) to explore the changes in metabolites and related metabolic pathways induced by MVA in HaCaT cells. This suggests that MVA treatment substantially alters the cellular metabolic state. The boxplot analysis indicates that MVA altered the levels of several metabolites, including 18-beta-Glycyrrhetinic acid, Palmitoylethanolamide (PEA), 17-AAG, l-arginine, and Dehydroepiandrosterone (DHEA), all of which exhibit significant biological effects in anti-aging, anti-inflammatory, and antioxidant properties. Additionally, key metabolic pathways, such as Arginine and proline metabolism, Purine metabolism, and Arachidonic acid metabolism, also highlighted potential anti-aging pathways of MVA. Our findings suggest that MVA may improve skin health through multiple metabolic mechanisms, supporting its potential as an anti-aging agent.

**Supplementary Information:**

The online version contains supplementary material available at 10.1038/s41598-025-25545-9.

## Introduction

Skin aging is a complex and inevitable process influenced by both intrinsic factors and external environmental conditions, causing structural and functional changes in the skin. Intrinsic aging results from natural physiological processes like genomic instability, cellular senescence, and telomere shortening, leading to decreased antioxidant capacity and increased reactive oxygen species (ROS). In contrast, extrinsic aging is primarily shaped by environmental factors, including ultraviolet (UV) radiation (photoaging), pollution, smoking, alcohol consumption, and unhealthy lifestyles. These factors contribute to increased skin roughness, deeper wrinkles, irregular pigmentation, and the formation of age spots^1,2^^.^

Anti-aging skin treatments have gained significant attention, focusing on improving skin health and appearance through various strategies. One effective approach is the topical application of antioxidants, such as vitamins C, B3, and E, as well as polyphenols and flavonoids, which help combat skin aging by neutralizing free radicals—especially important for addressing photoaging-induced damage. Furthermore, the retinoid family is recognized as one of the most promising active ingredients for anti-aging treatments. The anti-wrinkle effects of retinoid-containing topical formulations are attributed to their ability to promote keratinocyte proliferation, thicken the epidermis, regulate collagen homeostasis, enhance the epidermal barrier, reduce transepidermal water loss (TEWL), and modulate metalloproteinase activity^3,4^.

However, the use of retinoids faces several challenges. Retinoic acid (RA), also known as tretinoin, is the metabolically active form of vitamin A (VA) responsible for its ultimate biological effects^5^. Due to its potent skin-irritating properties at high doses, retinoic acid is restricted to prescription use in dermatological treatments and is prohibited in cosmetic formulations. In contrast, retinol and retinyl esters are the dietary forms commonly referred to as vitamin A^6^. Retinyl esters exhibit significantly higher tolerability on human skin than retinoic acid, making them widely used in cosmetic formulations to reduce wrinkles, fade pigmentation, and improve acne. However, exogenous retinyl esters must be enzymatically converted on the skin surface into retinoic acid in order to bind to receptors and exert their biological activity. This conversion is limited by enzyme activity, local delivery, and stability, resulting in reduced efficacy, even though retinyl esters are generally better tolerated by the skin^7^.

Improper or excessive use of topical retinol (RET) may result in potential side effects, such as dryness, redness, and peeling of the skin, which can cause discomfort^8^. Studies have shown that Chinese individuals exhibit heightened sensitivity to irritation reactions caused by retinol and retinoid treatments compared to Caucasians. This sensitivity manifests as erythema, desquamation, dryness, and elevated transepidermal water loss (TEWL), leading to temporary disruption of the skin barrier. These effects render conventional retinol treatments less suitable for Chinese individuals^9^. There are other retinol-based compounds available on the market, such as hydroxypinacolone retinoate (HPR), however, these compounds have certain drawbacks, such as reduced efficacy and other disadvantages.

Therefore, the development of novel vitamin A derivatives that integrate stability, safety, and potent anti-aging properties is not only crucial for overcoming the limitations of existing ingredients but also for driving advancements in advancing the fields of anti-aging and skin repair to new heights. The Magic vitamin A (MVA) derivatives were designed and synthesized by Coachchem. This study aims to evaluate the potential biological functions of MVA in skin health management and skin aging through metabolomics analysis.

## Results

### Computational studies on Magic Vitamin A (MVA) and *iso*-MVA

**Fig. 1:**
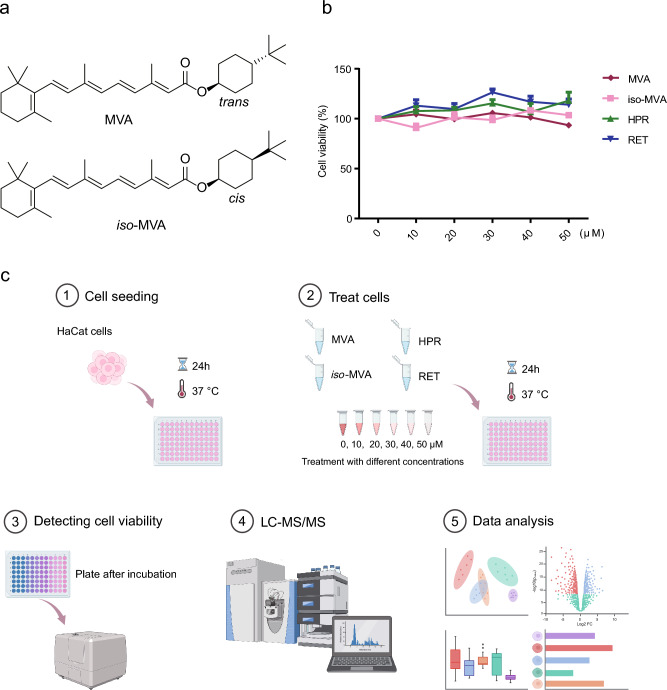
The molecular formula of vitamin A derivatives and MTT cytotoxicity assay analysis of cell viability. (**a**) The molecular formula of vitamin A derivatives named MVA; (**b**) HaCat cells were treated with different concentrations of MVA, *iso*-MVA, HPR, Retinol (5,10, 15, 20, 25, 30, 40 and 50 μM), respectively. MTT cytotoxicity assay analysis of cell viability; (**c**) Scheme of the experiment design.

The RAR crystallographic structure (PDB ID: 6EU9) with retinoic acid (RA) as inhibitor was obtained from the Protein Data Bank. The target protein was pre-processed using the Protein Preparation Wizard in the Schrödinger suite. The docking studies were using Schrodinger Glide standard precision (SP) scoring function. We used this self-docking approach to determine which docking conformations performed best at reproducing experimentally determined inhibitor poses during docking. To better clarify the difference in activity between different compounds (MVA and *iso*-MVA), molecular docking study was carried out. The molecular formula of MVA and *iso*-MVA is shown in Fig. 1a. In one category, the MVA could be elongated into the active site of RAR receptor, while there was no hydrogen bonds generated from MVA with the active site of RAR. Interestingly, there is no hydrogen bonds formed with residues around active site, and there are less residues form hydrophobic and van der Waals interactions. As shown in Fig. S1, the MVA participates in van der Waals forceswith Leu394, Ile397, Phe414, and Val356 of active site. As showed in Fig. S2, the *iso*-MVA can well interact with residues Ile398, Ile397, Arg355, and Leu359 by forming protein–ligand van der Waals contacts, electrostatic interactions. As showed in Fig. S3, the MVA could contribute to forming van der Waals forces and strong hydrophobic interactions with some key hydrophobic residues in the active site, such as Thr233, Ser229, Ile236, Ser232, and Phe228, etc. As showed in Fig. S4, the Predicted binding mode of *iso*-MVA within the binding pocket of RAR, which includes protein–ligand van der Waals contacts, electrostatic interactions. The *iso*-MVA could contribute to forming van der Waals forces and strong hydrophobic interactions with some key hydrophobic residues in the active site, such as Leu269, Ser232, Thr233. As showed in Fig. S5, the MVA could contribute to forming van der Waals forces and strong hydrophobic interactions with some key hydrophobic residues in the active site, such as Val270, Phe351, Phe444, Phe351, and His440, etc. As showed in Fig. S6, predicted binding mode of *iso*-MVA within the binding pocket of RAR, which includes protein–ligand van der Waals contacts, electrostatic interactions. The *iso*-MVA could contribute to forming van der Waals forces and strong hydrophobic interactions with some key hydrophobic residues in the active site, such as Phe444, Cys437, Val347. As showed in Fig. S7, predicted binding mode of MVA within the binding pocket of RAR, which includes protein–ligand van der Waals contacts, electrostatic interactions. The MVA could contribute to forming van der Waals forces and strong hydrophobic interactions with some key hydrophobic residues in the active site, such as Ile396, Ser232, Val395. As showed in Fig. S8, the *iso*-MVA could contribute to forming van der Waals forces and strong hydrophobic interactions with some key hydrophobic residues in the active site, such as Ser232, Leu305, Val395. Therefore, molecule docking analysis may conveniently explain why MVA have better potency than *iso*-MVA. Comparing several forms of VA and their derivatives, MVA demonstrates a superior performance in promoting collagen synthesis, exhibiting nearly an order of magnitude higher efficacy than the other compounds.

### Metabolomic classification and principal component analysis

**Fig. 2:**
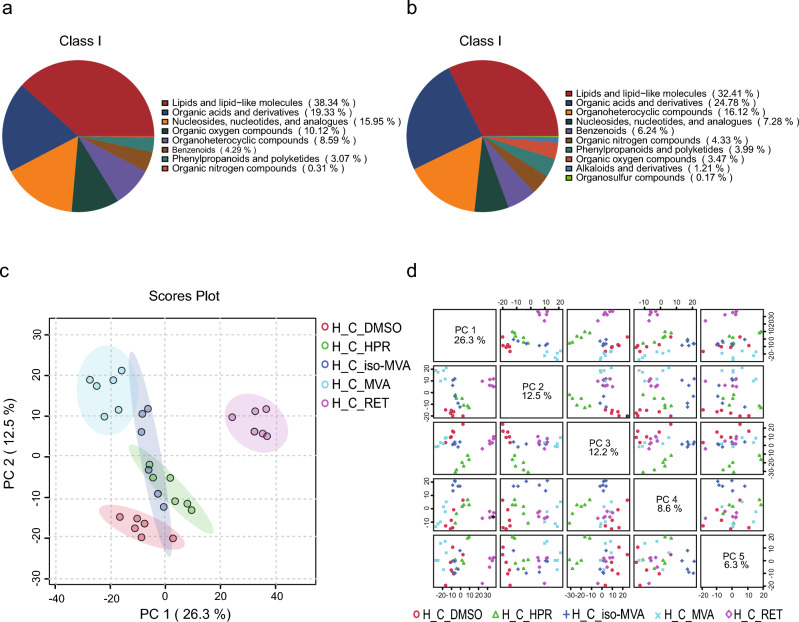
Metabolite classification and principal component analysis. (**a**) Pie chart of metabolite class I classification in positive ion mode; (**b**) Pie chart of metabolite class I classification in negative ion mode. (**c**) PCA scores plot of five groups. PERMANOVA results: R-squared: 0.90523; p-value (based on 999 permutations): 0.001. (**d**) pairwise score plot for top 5 PCs.

As can be seen from Fig. 1b, cell viability treated with MVA, *iso*-MVA, HPR and RET are almost at the same level, indicating that MVA, *iso*-MVA, HPR and RET have no cytotoxicity and can be used for treatment. Metabolomics analysis included a total of 30 samples from four experimental groups (H_C_MVA, H_C_*iso*-MVA, H_C_HPR, H_C_RET) and one control group (H_C_DMSO) (Fig. 1c). A total of 971 metabolites were identified in the positive ion mode and 409 metabolites in the negative ion mode (detailed results available in Supplementary Table S1). The chemical classification of identified metabolites was statistically analyzed, and a pie chart of Class I metabolite classifications was created to reflect the distribution and count of metabolites in each category. As shown in Fig. 2a,b, among the identified metabolites in the positive ion mode, the five most abundant categories were Lipids and lipid-like molecules, organic acids and derivatives, organoheterocyclic compounds, nucleosides, nucleotides, and analogues, and benzenoids. In the negative ion mode, the five most abundant categories were Lipids and lipid-like molecules, organic acids and derivatives, nucleosides, nucleotides, and analogues, organic oxygen compounds, and organoheterocyclic compounds.

The metabolite data identified in the positive and negative ion modes were merged for further processing. The results of multivariate statistical analysis based on cellular metabolites are shown in Fig. 2c,d. We compared the H_C_MVA group with the H_C_DMSO, H_C_*iso*-MVA, H_C_HPR, and H_C_RET groups. The unsupervised PCA revealed a clear separation trend between the H_C_MVA group and the other four groups (Fig. 2c). Additionally, some overlap was observed between the H_C_*iso*-MVA group and clusters such as H_C_HPR (R-squared: 0.90523; p-value (based on 999 permutations): 0.001).

### Multivariate statistical analysis

This study primarily focuses on the similarities and differences in metabolites between the H_C_MVA group and the other four groups, thus a detailed analysis was conducted comparing the metabolites of the H_C_MVA group with those of the other four groups.

**Fig. 3:**
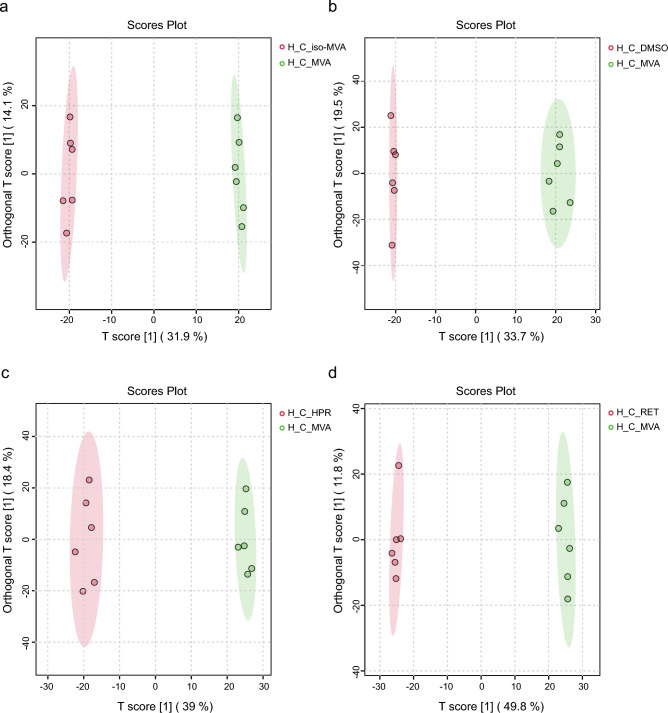
Multivariate statistical analysis of H_C_MVA. (**a**) OPLS-DA scores plot of H_C_MVA and H_C_ *iso*-MVA (R^2^X = 0.561, R^2^Y = 0.999, Q^2^ = 0.983). (**b**) OPLS-DA scores plot of H_C_MVA and H_C_DMSO(R^2^X = 0.532, R^2^Y = 0.997, Q^2^ = 0.949). (**c**) OPLS-DA scores plot of H_C_MVA and H_C_HPR(R^2^X = 0.574, R^2^Y = 0.996, Q^2^ = 0.971). (**d**) OPLS-DA scores plot of H_C_MVA and H_C_RET(R^2^X = 0.616, R^2^Y = 0.999, Q^2^ = 0.983).

The OPLS-DA model analysis revealed a clear separation between the H_C_MVA group and the other four groups, indicating significant changes in the cellular metabolic state of the H_C_MVA group. As shown in Fig. 3a–d, distinct clustering was observed among the groups: (H_C_MVA group vs. H_C_ *iso*-MVA group: R^2^X = 0.561, R^2^Y = 0.999, Q^2^ = 0.983; H_C_MVA group vs. H_C_DMSO group: R^2^X = 0.532, R^2^Y = 0.997, Q^2^ = 0.949; H_C_MVA group vs. H_C_HPR group: R^2^X = 0.574, R^2^Y = 0.996, Q^2^ = 0.971; H_C_MVA group vs. H_C_RET group: R^2^X = 0.616, R^2^Y = 0.999, Q^2^ = 0.983).

### Differential metabolite analysis

**Fig. 4:**
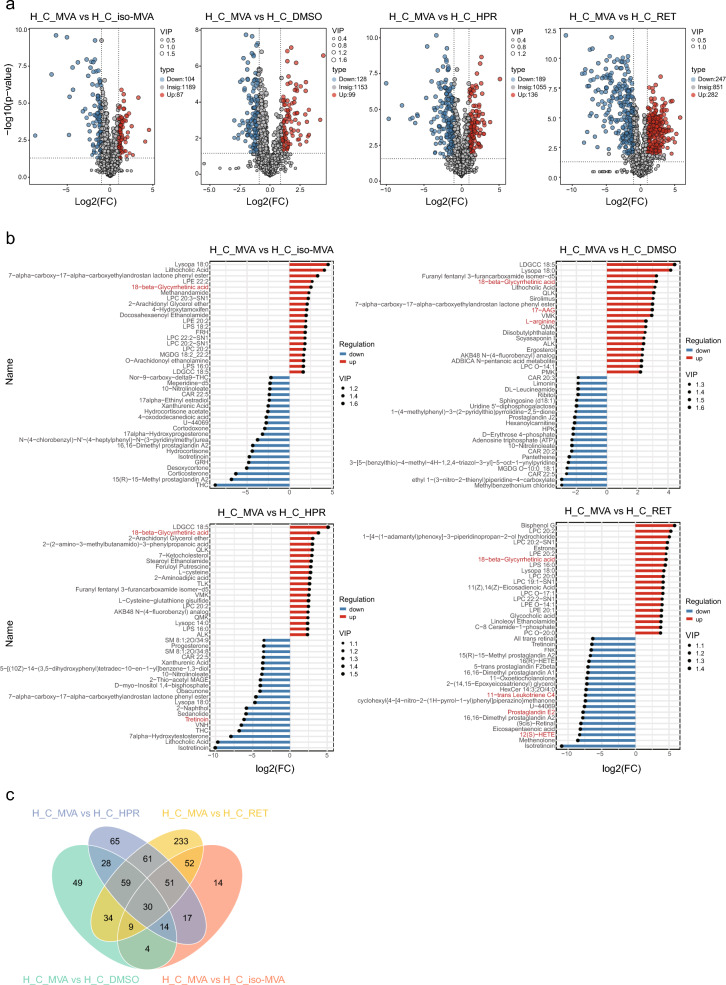
Differential Metabolite Analysis of H_C_MVA. (**a**) Volcano plot for H_C_MVA vs H_C_*iso*-MVA, H_C_MVA vs H_C_DMSO, H_C_MVA vs H_C_HPR and H_C_MVA vs H_C_RET. Blue indicates downregulated differential metabolites in H_C_MVA, and red indicates upregulated differential metabolites in H_C_MVA. Points with VIP greater than 1 based on the OPLS-DA model are colored in the plot (VIP > 1.0, Log2(FC) ≥ 1, P-value < 0.05). (**b**) Lollipop chart for H_C_MVA vs H_C_*iso*-MVA, H_C_MVA vs H_C_DMSO, H_C_MVA vs H_C_HPR and H_C_MVA vs H_C_RET. The top 20 differential metabolites with the highest absolute fold change values are displayed. Blue indicates downregulated differential metabolites in H_C_MVA, and red indicates upregulated differential metabolites in H_C_MVA. VIP values are represented by the size of the points. (**c**) Venn diagram of differential metabolites. The numbers in the diagram represent the count of overlapping differential metabolites among the groups or the unique differential metabolites in each group.

Differential metabolites were screened based on three parameters: VIP, FC, and p-value (VIP > 1.0, FC > 2 or FC < 0.5, and p-value < 0.05) (Fig. 4a, Supplementary Table S2). A total of 191 significantly different metabolites were identified between the H_C_MVA and H_C_*iso*-MVA groups, with 87 metabolites significantly upregulated and 104 significantly downregulated. Between the H_C_MVA and H_C_DMSO groups, 227 significantly different metabolites were identified, including 99 upregulated and 128 downregulated metabolites; Between the H_C_MVA and H_C_HPR groups, 325 significantly different metabolites were identified, including 136 upregulated and 189 downregulated metabolites; Between the H_C_MVA and H_C_RET groups, 529 significantly different metabolites were identified, including 282 upregulated and 247 downregulated metabolites.

The lollipop plot highlighted the top 20 upregulated and downregulated differential metabolites ranked by |log2FC| (Fig. 4b): Between the H_C_MVA and H_C_*iso*-MVA groups, the most upregulated metabolites with large |log2FC| values included Lithocholic Acid, 18-beta-Glycyrrhetinic acid, Methanandamide, 2-Arachidonyl Glycerol ether, 4-Hydroxytamoxifen, and Docosahexaenoyl Ethanolamide, while the most downregulated metabolites included THC, Corticosterone, Desoxycortone, Isotretinoin, and Hydrocortisone; Between the H_C_MVA and H_C_DMSO groups, the most upregulated metabolites with large |log2FC| values included 18-beta-Glycyrrhetinic acid, Lithocholic Acid, 17-AAG, l-arginine, and Soyasaponin I, while the most downregulated metabolites included Pantetheine, 10-Nitrolinoleate, Adenosine triphosphate (ATP), D-Erythrose 4-phosphate, Hexanoylcarnitine, and Prostaglandin J2; Between the H_C_MVA and H_C_HPR groups, the most upregulated metabolites included 18-beta-Glycyrrhetinic acid, 2-Arachidonyl Glycerol ether, 7-Ketocholesterol, Feruloyl Putrescine, and Stearoyl Ethanolamide, while the most downregulated metabolites included Isotretinoin, Lithocholic Acid, 7alpha-Hydroxytestosterone, THC, and Tretinoin; Between the H_C_MVA and H_C_RET groups, the most upregulated metabolites included Estrone, 18-beta-Glycyrrhetinic acid, Glycocholic acid, Linoleoyl Ethanolamide, and l-cysteine, while the most downregulated metabolites included Isotretinoin, Methenolone, 12(S)-HETE, Eicosapentaenoic acid, (9cis)-Retinal, and Prostaglandin E2.

Based on the Venn diagram of differential metabolites, the overlapping metabolites and unique differential metabolites between each pair of groups can be observed (Fig. 4c): The H_C_MVA and H_C_*iso*-MVA groups had 14 unique differential metabolites; The H_C_MVA and H_C_DMSO groups had 49 unique differential metabolites; The H_C_MVA and H_C_HPR groups had 65 unique differential metabolites; The H_C_MVA and H_C_RET groups had 233 unique differential metabolites; Across all four control groups, there were 30 common differential metabolites.

### Effective metabolite analysis

**Fig. 5:**
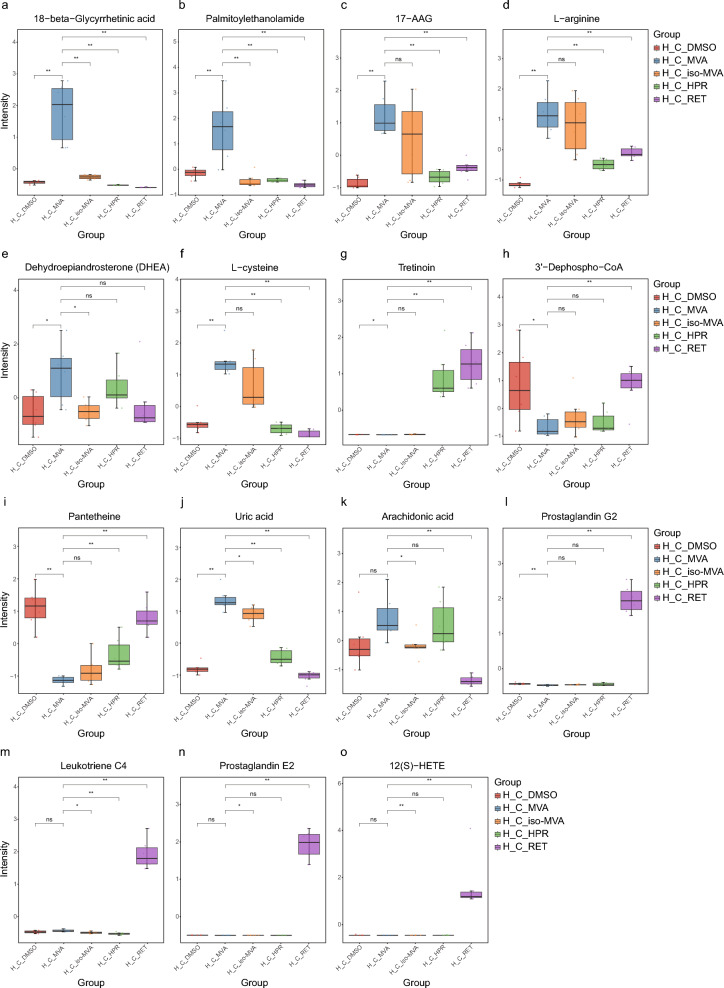
Analysis of the intensity of selected key metabolites. (**a**) Boxplot of 18-beta-Glycyrrhetinic acid. When the P-value > 0.05, it is represented as “ns”; when the P-value < 0.05, it is represented as "*"; and when the P-value < 0.01, it is represented as "**". (**b**) Boxplot of Palmitoylethanolamide (PEA). (**c**) Boxplot of 17-AAG. (**d**) Boxplot of l-arginine. (**e**) Boxplot of dehydroepiandrosterone (DHEA). (**f**) Boxplot of l-cysteine. (**g**) Boxplot of Tretinoin. (**h**) Boxplot of 3′-Dephospho-CoA. (**i**) Boxplot of Pantetheine. (**j**) Boxplot of Uric acid. (**k**) Boxplot of arachidonic acid. (**l**) Boxplot of prostaglandin G2. (**m**) Boxplot of Leukotriene C4. (**n**) Boxplot of prostaglandin E2. (**o**) Boxplot of 12(S)-HETE.

A subset of metabolites with potential anti-aging or anti-inflammatory effects, such as 18-beta-Glycyrrhetinic acid, PEA, 17-AAG, l-arginine, DHEA, and Tretinoin, as well as metabolites with key roles in pathways, including l-cysteine, 3′-Dephospho-CoA, Pantetheine, Uric acid, Arachidonic acid, Prostaglandin G2, Leukotriene C4, Prostaglandin E2, and 12(S)-HETE, were selected, and their boxplots were generated (Fig. 5a–o). In the H_C_MVA group, 18-beta-Glycyrrhetinic acid and PEA levels were significantly higher than in the other four groups.17-AAG and l-arginine levels in the H_C_MVA group were significantly higher than in the H_C_DMSO, H_C_HPR, and H_C_RET groups, and slightly higher than in the H_C_*iso*-MVA group, but without significant differences; DHEA levels in the H_C_MVA group were higher than in the other four groups, but the differences were not significant; Tretinoin levels in the H_C_HPR and H_C_RET groups were significantly higher than in the other three groups.

### Metabolic pathway analysis

**Fig. 6:**
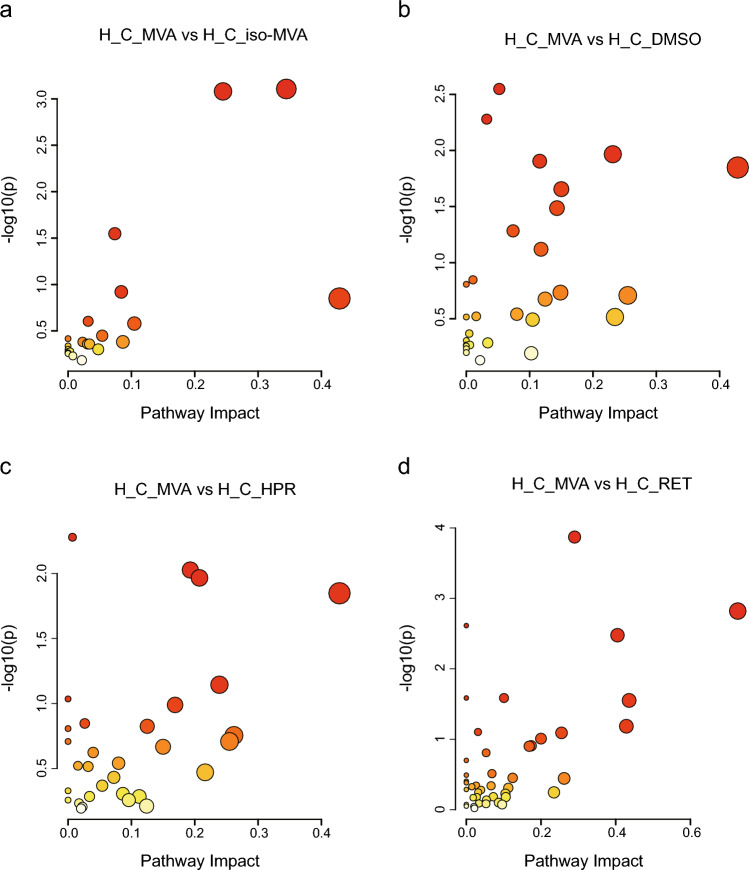
Metabolic pathway analysis of H_C_MVA. (**a**) Metabolic pathway topology of H_C_MVA and H_C_*iso*-MVA. The y-axis represents the − log (p) value of the pathway enrichment analysis, and the x-axis represents the pathway impact value of topology analysis. (**b**) Metabolic pathway topology of H_C_MVA and H_C_DMSO. (**c**) Metabolic pathway topology of H_C_MVA and H_C_HPR. (**d**) Metabolic pathway topology of H_C_MVA and H_C_RET.

The differential metabolites between the H_C_MVA group and the other four control groups were imported into MetaboAnalyst 6.0 for pathway analysis. The significance of pathway changes was calculated using topology-based metabolic pathway analysis (Fig. 6a–d): Pathways enriched (P < 0.05) between the H_C_MVA and H_C_*iso*-MVA groups included: Tryptophan metabolism, Steroid hormone biosynthesis, and Glycerophospholipid metabolism; Pathways enriched between the H_C_MVA and H_C_DMSO groups included: Purine metabolism、Pantothenate and CoA biosynthesis、Pyrimidine metabolism、Taurine and hypotaurine metabolism、Biotin metabolism、Lysine degradation; Pathways enriched between the H_C_MVA and H_C_HPR groups included: Purine metabolism、Arginine and proline metabolism、Pantothenate and CoA biosynthesis、Taurine and hypotaurine metabolism; Pathways enriched between the H_C_MVA and H_C_RET groups included: Steroid hormone biosynthesis、Retinol metabolism、Biosynthesis of unsaturated fatty acids、Pantothenate and CoA biosynthesis、Linoleic acid metabolism、Sphingolipid metabolism、Arachidonic acid metabolism. (Detailed information on the metabolic pathways significantly enriched by the differential metabolites is provided in Supplementary Table S3).

## Discussion

Skin aging affects the structure and function of the epidermis, the epidermal-dermal junction, and the dermis, manifested by a decline in cellular proliferation, a reduction in epidermal thickness, flattening of the basement membrane, and changes in the extracellular matrix (ECM) components of the dermis, such as the degeneration of collagen and elastin fibers. Ultimately, this leads to thinning of the skin, decreased elasticity, and deepening of wrinkles^10,11^. Our study explores the potential of various metabolites produced in the H_C_MVA group to counteract these aging effects.

The metabolites produced in the H_C_MVA group, including 18-beta-Glycyrrhetinic acid, PEA, 17-AAG, l-arginine, and DHEA, all demonstrate potential anti-aging benefits. Meanwhile, in the significantly enriched pathways, the H_C_MVA group also exhibits certain anti-aging effects. 18-beta-Glycyrrhetinic acid is the main active compound extracted from licorice, known for its various biological effects^12^. It has been shown to improve skin appearance, increase collagen content, and prevent UV-induced structural damage by inhibiting the expression of Matrix Metalloproteinases (MMPs), which are key mediators of collagen degradation^13,14^. PEA, a member of the FAEs family, has potential benefits in treating various skin disorders, including contact allergic dermatitis, atopic dermatitis, eczema, acne, seborrhea, and pain and pruritus^15,16^. 17-AAG, as an Hsp90 (Heat Shock Protein 90) inhibitor, has primarily been studied in the context of cancer and tumor-related diseases^2,17,18^. It has also demonstrated potential in treating cutaneous leishmaniasis by clearing Leishmania parasites and reducing inflammatory mediators^19^. l-arginine, a conditionally essential amino acid, promotes wound healing, enhances collagen synthesis, and improves cutaneous vascular reflex dilation in the elderly^20–22^. DHEA, a natural steroid hormone, improves skin condition by enhancing hydration, increasing epidermal thickness, regulating sebaceous balance, and stimulating collagen synthesis^23,24^.

Additionally, our results show that the metabolites in the H_C_MVA group are significantly enriched in proline metabolism, purine metabolism, and the biosynthesis pathways of pantothenate and CoA. As hydroxyproline is a key substrate for collagen synthesis, it may influence collagen production through this pathway^25^. Adenosine in the purine metabolism pathway interacts with A1 and A2A adenosine receptors in the skin, promoting collagen synthesis and improving skin condition^26^. CoA promotes skin repair by maintaining mitochondrial function, thereby delaying skin aging^27^. The differential metabolites between the H_C_MVA and other groups suggest that these pathways play crucial roles in skin health and aging.

Inflammation and oxidative stress are key factors in skin aging and various skin disorders^28^. Senescent keratinocytes and fibroblasts can influence the skin microenvironment by secreting inflammatory cytokines and extracellular matrix-modifying enzymes, thereby accelerating skin aging^29–31^. The metabolites produced in the H_C_MVA group, such as 18-beta-Glycyrrhetinic acid, PEA, 17-AAG and l-arginine, all demonstrate significant anti-inflammatory effects. 18-beta-Glycyrrhetinic acid inhibits the production of pro-inflammatory cytokines and reduces oxidative stress^32^. PEA has demonstrated significant effects in controlling inflammation and pain^33^. Furthermore, Singh et al. reported that the topical application of 17-AAG may help prevent UV-induced inflammation and skin squamous cell carcinoma^34^. The availability, synthesis, and metabolism of l-arginine are key factors in immune responses, and numerous studies have shown that it can reduce inflammation^35,36^. DHEA has also demonstrated significant anti-inflammatory effects in various mouse inflammation models^37,38^, suggesting its potential value as an anti-inflammatory supplement. Furthermore, the differential metabolites between the H_C_MVA and H_C_*iso*-MVA groups were significantly enriched in the tryptophan metabolism pathway. These tryptophan derivatives promote the expression of terminal differentiation proteins (such as Filaggrin and keratins) by activating the AhR/ARNT signaling pathway, thereby maintaining the skin barrier and modulating immune responses^39^. Previous studies have found that tryptophan metabolites are significantly reduced in the skin lesions of atopic dermatitis^40^, highlighting the important role of the tryptophan metabolism pathway in immune regulation.

The skin is frequently exposed to damage from exogenous factors such as UV radiation, pollution, and mechanical stress, as well as endogenous factors like cellular metabolism and enzymatic reactions. However, it maintains a balance with ROS and free radicals through antioxidant defense mechanisms^41,42^. The metabolites produced in the H_C_MVA group, such as 18-beta-Glycyrrhetinic acid, l-arginine, purine metabolism and l-cysteine, all show significant antioxidant effects. 18-beta-Glycyrrhetinic acid, as an effective antioxidant, can inhibit oxidative stress^43^, prevent oxidative DNA fragmentation^44^. l-arginine also contributes to the activation of endogenous antioxidant defenses. Liang et al. were the first to demonstrate the impact of l-arginine on the activation of endogenous antioxidant defenses^45^. l-cysteine is a multifunctional amino acid that plays a crucial role in antioxidant defense, collagen synthesis, energy metabolism, and skin health. l-cysteine helps maintain cellular function and homeostasis by regulating redox balance and metabolic pathways^46^. Studies have shown that increasing the supply of l-cysteine or its precursors can significantly promote the synthesis of GSH^47^. However, while the synthesis pathway of glutathione was enriched in these groups, it was not significant. Moreover, we believe that the primary metabolites produced in the H_C_MVA group have minimal irritation potential on the skin, making them potentially safer for topical application and more suitable for individuals with sensitive skin.

Retinol and retinoid-like compounds (e.g., HPR) are widely used for their anti-aging benefits but often cause skin irritation^48^. These products exert their effects on the skin through conversion to Tretinoin, and our results also show that the Tretinoin levels in the H_C_RET and H_C_HPR groups are significantly higher than in the other three groups. These side effects not only affect patient comfort but may also interfere with treatment adherence, posing a significant challenge in clinical use. However, the active metabolites produced in the H_C_MVA group are relatively mild, with some of them, such as PEA, having been shown to be non-irritating and non-sensitizing to human skin. The differential metabolites between the H_C_MVA and H_C_RET groups suggest that the H_C_MVA group may promote a transition from a pro-inflammatory to an anti-inflammatory phase, reducing the production of inflammatory metabolites. This indicates that MVA products may offer similar anti-aging benefits with fewer side effects. Although PEA has demonstrated excellent therapeutic effects, its bioavailability is significantly limited due to poor water solubility, low stability, and difficulty in penetrating the stratum corneum barrier^18^. Therefore, approaches such as emulsification^49^, micronization^50^, or advanced delivery systems^18,33^ are commonly employed to enhance its bioavailability and functionality. Accordingly, MVA products not only promote the production of multiple active metabolites in HaCaT cells, enabling these compounds to exert synergistic effects, but also partially improve the bioavailability of certain metabolites, such as PEA.

## Conclusion

In conclusion, the multiple metabolites produced by MVA (e.g., 18-beta-Glycyrrhetinic acid, PEA, 17-AAG, l-arginine, and DHEA) contribute to anti-aging, anti-inflammatory, and antioxidant activities. MVA may regulate various metabolic pathways, such as arginine and proline metabolism, purine metabolism, and arachidonic acid metabolism, which collectively promote collagen synthesis, reducing elastic fiber degradation, repairing skin damage, and modulating oxidative stress and inflammatory responses. These mechanisms effectively improve skin structure and function, slowing the skin aging process. Additionally, compared to the H_C_RET (retinol) and H_C_HPR (retinoid-like) groups, the effective metabolites produced in the H_C_MVA group exhibit a milder effect. Compounds such as PEA have been demonstrated to be non-irritating to the skin, making MVA potentially more suitable for individuals with sensitive skin. Although certain metabolites, like PEA, have low bioavailability, their efficacy may be enhanced through metabolic conversion by MVA within cells. Therefore, MVA products hold substantial promise in skin health management, offering multiple benefits such as anti-aging, anti-inflammatory, and antioxidant effects. With their gentler profile, MVA-based products are well-suited for a wide range of skincare needs.

## Materials and methods

### RAR structure collation and selection

The RAR crystallographic structure (PDB ID: 6EU9) with retinoic acid (RA) as inhibitor was obtained from the Protein Data Bank. The target protein was pre-processed using the Protein Preparation Wizard in the Schrödinger suite. This process involved assigning bond orders, zero order bonds to metal atoms, selenomethionine to methionine conversion, filling absent hydrogens, capping termini, side chains, and loops, and removing waters beyond 5 Å distance surrounding the co-crystallized ligand. All water molecules that did not contribute to the binding interactions were removed. The hydrogen bonds of the protein were optimized to refurbish the superimposing hydrogen atoms and minimized using the OPLS-3 force field with a root mean square deviation (RMSD) value of 0.30 Å. All non-protein atoms were removed from target protein, with the exception of the natural ligand. The protein grid was generated using the Receptor Grid Generation module of Schrödinger. In the receptor grid generation module, the grid is defined by selecting the RA ligand that constitutes the binding pocket of target protein. The atoms of the target protein were fixed within default parameters of the Van der Waals radius scaling factor of 1 Å with a partial charge cutoff of 0.3 Å using the OPLS-3 force field. We used the LigPrep module of the Schrodinger suite to prepare each ligand for docking. LigPrep was conFig.d to exhaustively generate all possible tautomers and stereoisomers for every input molecule. All other target compounds that were evaluated in this study came from existing SDF files that The provided three-dimensional structural information for each molecule in this study came from SDF files and used EPIK to assign ligand protonation states at value of 7.0 ± 2.0.

### Molecular docking methodology

Docking was done using Glide software (Schrodinger suite, version 2015). The receptor was prepared via the Protein Preparation Wizard to refine bond orders, fill in missing side chains and loops, add and optimize hydrogen bond networks, and assign force-field atom types. Protein minimization was carried out using the OPLS4 force field. Epik was employed to generate the het states at pH 7.4, and PROPKA was used to generate the protonation states of residues. The LigPrep module was used to prepare ligands, including generation of protonation states and generating stereoisomers and tautomers. Docking was performed in the Standard Precision (SP) scoring mode. We also performed molecular docking using human homolog structures (PDB IDs: 3KMZ, 3A9E, and 1DKF), and obtained comparable results, further supporting the reliability of our findings. The docking studies were using Schrodinger Glide standard precision (SP) scoring function. We used this self-docking approach to determine which docking conformations performed best at reproducing experimentally determined inhibitor poses during docking. Performance was assessed by the calculation of the in-place root-mean-square deviation (rmsd) of the lowest scoring docked pose to the experimental position. The lower the in-place rmsd, the higher we considered the predictive accuracy to be for a model. We chose to use Glide SP to perform molecular docking in this study. Glide SP uses a series of hierarchical filters to exhaustively sample the conformational, orientational, and positional landscape for each ligand. The docked pose was deterministically returned after a final geometry optimization was performed using the OPLS-3 force field. Receptor grids were generated by selecting the co-crystallized inhibitor in the Maestro workspace for each protein structure. We used the center of the molecule to define the center of the receptor grid box. This allowed the centroids of any docked molecules to sample a 10 × 10 × 10 Å inner search space, while the periphery of each molecule could extend 20 Å in any direction. Any residues that could freely rotate within the receptor grid were allowed to do so. We opted to use flexible ligand sampling to consider the effects of stereoisomers, alternative ring conformers, or pyramidal nitrogen inversions when these options were possible. In-place rmsds were calculated by comparing the positions of all heavy atoms in the docked pose to the input coordinates without superimposition.

Building upon our previous work, we have successfully synthesized the following two novel vitamin A derivatives. [Refer to CN 115925666 B].

### Molecular docking studies

To better clarify the difference in activity between different compounds (MVA and *iso*-MVA), molecular docking study was carried out. In one category, the MVA could be elongated into the active site of RAR receptor, while there was no hydrogen bonds generated from MVA with the active site of RAR. The ester moiety in MVA does not participate in hydrogen bonding with residues in target protein. The majority of contacts made by RAR with MVA were weak van der Waals interactions, and the carboxylate moiety of the ligand did not form any noticeable hydrogen bonds with surrounding RAR residues and only form hydrophobic and van der Waals interactions with residues around the active site, such as Val523, Phe414, Leu398 and Phe430. Also in other category, the *iso*-MVA could be elongated into the active site of RAR receptor. Interestingly, there is no hydrogen bonds formed with residues around active site, and there are less residues form hydrophobic and van der Waals interactions. Therefore, molecule docking analysis may conveniently explain why MVA have better potency than *iso*-MVA.

### Comparison of molecular effects

Comparing several forms of VA and their derivatives, MVA demonstrates a superior performance in promoting collagen synthesis, exhibiting nearly an order of magnitude higher efficacy than the other compounds.

### Cell treatment and metabolite extraction

HaCaT cells were seeded into 96-well plates (Costar, USA) at a density of 1 × 10^4^ cells per well in 100 μL of medium. After cell attachment, cells were treated with different concentrations (5, 10, 15, 20, 25, 30, 40, and 50 μM) of MVA, *iso*-MVA, HPR, or Retinol, and were incubated for 24 h. The control group and the blank group were added with the same amount of culture medium with and without cells respectively, with 6 repeats in each group. After a period of 24 h incubation at 37 °C in 5% CO_2_, cell viability was assayed with a MTT kit (Beyotime, Haimen, China) 24 h later, the prepared tetramethylazolium salt solution was diluted to 0.5 mg/mL with a medium, then 100 μL formazan was added to each well 4 h later, wrapped in tin foil and vortexed, RP absorption (570 nM) was measured by enzyme labeling, and cell survival was calculated^51^.

Cells achieved 80% confluency after 48 h incubation in 10 cm dishes (Corning, USA). MVA, *iso*-MVA, HPR and Retinol were administered to final concentrations of 30 μM by adding 10 μL of 30 mM of MVA, *iso*-MVA, HPR and Retinol to 10mL of medium. A control group was used which involved the application of DMSO (sigma, China). Sixth replicates were done for each exposure group and the control. After exposure, the medium was discarded and cells were washed twice with 10mL room temperature phosphate buffer solution (PBS) (servicebio, China). 1 × 10^7^ cells were collected from each group in 15 mL centrifuge tube (Corning, USA), After centrifuge for 5 min at 400 g, discard the supernatant and the cell precipitate were frozen in liquid nitrogen. Then the cell precipitate were transferred to a − 80 °C freezer until extracted and assayed.

The LC–MS/MS analysis of metabolites was entrusted to Beijing Novogene Technology Co., Ltd. Cell samples were placed in EP tubes, and 300 μL of 80% methanol–water solution was added before freezing in liquid nitrogen for 5 min. After thawing on ice, the samples were vortexed for 30 s, followed by ultrasonic treatment for 6 min. The samples were then centrifuged at 5000 rpm, 4 °C for 1 min. The supernatant was collected and transferred to a new centrifuge tube for lyophilization to prepare a dry powder. Equal volumes of 10% methanol–water solution were added to re-dissolve the sample, which was then used for LC–MS analysis. Quality control (QC) samples were prepared by pooling equal volumes of extracts from each sample and were interspersed throughout the experimental batch to assess the stability and reproducibility of the metabolomics analysis platform. Blank samples were prepared by replacing the biological material with 53% methanol–water solution and processed using the same pretreatment procedure to eliminate background ions.

### Chromatographic and mass spectrometric conditions

The analysis was performed using an Orbitrap Q Exactive™ HFX mass spectrometer (Thermo Fisher, Germany) coupled with a Vanquish UHPLC system. This combination of high-resolution mass spectrometry and liquid chromatography techniques enabled efficient and accurate sample analysis.

Samples from HaCaT cells were run in random order to prevent any run-dependent bias. Chromatographic separation was performed using a Hypersil Gold C18 column with a flow rate of 0.2 mL/min. Each sample was injected once. The injection volume was 5 μL in positive ion mode and 10 μL in negative ion mode. The mobile phase A consisted of 0.1% formic acid, and mobile phase B was methanol. Throughout the separation, the column was maintained at 40 °C, and the following gradient elution program was used to achieve separation: 0–1.5 min, 98% A, 2% B; 1.5–3 min, 15% A, 85% B; 3–10 min, 100% B; 10–12 min, 98% A, 2% B.

The mass spectrometer was equipped with an electrospray ionization (ESI) source, with the spray voltage set to 3.5 kV. The sheath gas flow rate was set to 35 psi, while the auxiliary gas flow rate and auxiliary gas heater temperature were set to 10 L/min and 350 °C, respectively. The ion transfer tube temperature (Capillary Temp) was maintained at 320 °C, and the S-lens RF level was set to 60. Both positive and negative ion modes (Polarity: positive, negative) were employed to ensure effective analysis of metabolites with different polarities. For MS/MS secondary scanning, the scan range was set to m/z 100–1500, using a data-dependent scan mode to enhance sensitivity and selectivity in the analysis.

### Data preprocessing and metabolite identification

The raw data files (.raw) were imported into the Compound Discoverer (CD) 3.3 software for preliminary processing. Each metabolite was subjected to basic screening based on parameters such as retention time and mass-to-charge ratio (m/z). Peak area correction was performed using the first QC sample to enhance identification accuracy. Parameters were set as follows: mass deviation at 5 ppm, signal intensity deviation at 30%, minimum signal intensity, and adduct ions for peak extraction. Peak area quantification was then conducted. Based on this, target ion information was integrated, and molecular formulas were predicted using molecular ion peaks and fragment ions.

Metabolites were matched against the mzCloud (https://www.mzcloud.org/), mzVault, and Masslist databases, and background ions were removed using blank samples. MzCloud and mzVault are both MS/MS-based level 2 spectral databases. However, since retention time (RT) was not matched during metabolite identification in this study, the identified metabolites can only be classified as MSI level 2. The Masslist database is a level 1 database based solely on accurate mass, and the corresponding metabolite identifications fall under MSI level 3 (detailed results available in Supplementary Table S1). The raw quantification results were normalized using the formula: Normalized Value = (Raw Quantification Value of Sample)/(Total Quantification Value of Metabolites in the Sample/Total Quantification Value of Metabolites in QC1 Sample), yielding relative peak areas. Compounds with a coefficient of variation (CV) greater than 30% in relative peak areas across QC samples were excluded. The final output included metabolite identification and relative quantification results. Identified metabolites were annotated using the KEGG database (https://www.genome.jp/kegg/pathway.html)^52–54^, HMDB database (https://hmdb.ca/metabolites), and LIPIDMaps database (http://www.lipidmaps.org/) to analyze their biological significance. All data processing was conducted on a Linux operating system (CentOS version 6.6) and analyzed using R and Python software.

### Statistical analysis

Median normalization was applied to the data to eliminate potential batch effects or processing errors among different samples, improving data consistency. Auto-scaling was conducted to remove inconsistencies in scale between variables. Principal Component Analysis (PCA) and Orthogonal Partial Least Squares Discriminant Analysis (OPLS-DA) were performed using the MetaboAnalystR package in R. PCA, an unsupervised multivariate statistical method, was used to reveal the original structure and trends in the metabolomics data. OPLS-DA, a supervised multivariate statistical method with pattern recognition capabilities, was employed to effectively eliminate irrelevant variation and identify differential metabolites. The cumulative R^2^Y and Q^2^ values were calculated to evaluate the model’s goodness-of-fit and predictive capability, providing a comprehensive measure of model performance. To assess the relative importance of each predictive variable in the OPLS-DA model, Variable Importance in Projection (VIP) values were calculated. Significant differences in metabolite concentrations between groups were tested using Student’s t-test (p < 0.05), and fold changes (FC) were calculated to quantify the differences between the two groups. Metabolic pathway analysis was conducted using the pathway analysis module of MetaboAnalyst 6.0 (https://www.metaboanalyst.ca).

## Supplementary Information

Supplementary Information 1.

Supplementary Information 2.

Supplementary Information 3.

Supplementary Information 4.

## Data Availability

The raw abundance table of metabolites can be found in Supplementary Table S1.

## References

[1] Quan, T. Human skin aging and the anti-aging properties of retinol. *Biomolecules.*10.3390/biom13111614 (2023). 10.3390/biom13111614 PMC10669284 38002296

[2] Zhang, Y. et al. Uncovering key mechanisms and intervention therapies in aging skin. *Cytokine Growth Factor Rev.* **79**, 66–80. 10.1016/j.cytogfr.2024.07.009 (2024). 39198086 10.1016/j.cytogfr.2024.07.009

[3] Kim, B. H. Safety evaluation and anti-wrinkle effects of retinoids on skin. *Toxicol. Res.* **26**, 61–66. 10.5487/tr.2010.26.1.061 (2010). 24278507 10.5487/TR.2010.26.1.061 PMC3834457

[4] Quan, T. et al. Retinoids suppress cysteine-rich protein 61 (CCN1), a negative regulator of collagen homeostasis, in skin equivalent cultures and aged human skin in vivo. *Exp. Dermatol.* **20**, 572–576. 10.1111/j.1600-0625.2011.01278.x (2011). 21488975 10.1111/j.1600-0625.2011.01278.x PMC3120908

[5] Mukherjee, S. et al. Retinoids in the treatment of skin aging: an overview of clinical efficacy and safety. *Clin. Interv. Aging* **1**, 327–348. 10.2147/ciia.2006.1.4.327 (2006). 18046911 10.2147/ciia.2006.1.4.327 PMC2699641

[6] Szymański, Ł. *et al.* Retinoic acid and its derivatives in skin. *Cells*. 10.3390/cells9122660 (2020). 10.3390/cells9122660 PMC7764495 33322246

[7] Sorg, O., Antille, C., Kaya, G. & Saurat, J. J. Retinoids in cosmeceuticals. *Dermatol. Ther.* **19**, 289–296. 10.1111/j.1529-8019.2006.00086.x (2006). 17014484 10.1111/j.1529-8019.2006.00086.x

[8] Carazo, A. et al. Vitamin A update: Forms, sources, kinetics, detection, function, deficiency, therapeutic use and toxicity. *Nutrients*10.3390/nu13051703 (2021). 34069881 10.3390/nu13051703 PMC8157347

[9] Goh, C. L., Tang, M. B., Briantais, P., Kaoukhov, A. & Soto, P. Adapalene gel 0.1% is better tolerated than tretinoin gel 0.025% among healthy volunteers of various ethnic origins. *J. Dermatol. Treat.* **20**, 282–288. 10.1080/09546630902763164 (2009). 10.1080/09546630902763164 19634042

[10] Blume-Peytavi, U. et al. Age-associated skin conditions and diseases: current perspectives and future options. *Gerontologist* **56**(Suppl 2), S230-242. 10.1093/geront/gnw003 (2016). 26994263 10.1093/geront/gnw003

[11] Csekes, E. & Račková, L. Skin aging, cellular senescence and natural polyphenols. *Int. J. Mol. Sci.*10.3390/ijms222312641 (2021). 34884444 10.3390/ijms222312641 PMC8657738

[12] Shinu, P. et al. Pharmacological features of 18β-glycyrrhetinic acid: a pentacyclic triterpenoid of therapeutic potential. *Plants*10.3390/plants12051086 (2023). 36903944 10.3390/plants12051086 PMC10005454

[13] Kong, S. Z. et al. The protective effect of 18β-Glycyrrhetinic acid against UV irradiation induced photoaging in mice. *Exp. Gerontol.* **61**, 147–155. 10.1016/j.exger.2014.12.008 (2015). 25498537 10.1016/j.exger.2014.12.008

[14] Fisher, G. J. et al. Mechanisms of photoaging and chronological skin aging. *Arch. Dermatol.* **138**, 1462–1470. 10.1001/archderm.138.11.1462 (2002). 12437452 10.1001/archderm.138.11.1462

[15] Petrosino, S. et al. Protective role of palmitoylethanolamide in contact allergic dermatitis. *Allergy* **65**, 698–711. 10.1111/j.1398-9995.2009.02254.x (2010). 19909294 10.1111/j.1398-9995.2009.02254.x

[16] Rao, A., Moussa, A. A., Erickson, J. & Briskey, D. Efficacy of topical palmitoylethanolamide (levagen+) for the management of eczema symptoms: a double-blind, comparator-controlled, randomized clinical trial. *Skin Pharmacol. Physiol.* **36**, 288–295. 10.1159/000536670 (2023). 38408443 10.1159/000536670 PMC10997259

[17] Newman, B., Liu, Y., Lee, H. F., Sun, D. & Wang, Y. HSP90 inhibitor 17-AAG selectively eradicates lymphoma stem cells. *Can. Res.* **72**, 4551–4561. 10.1158/0008-5472.Can-11-3600 (2012). 10.1158/0008-5472.CAN-11-3600 PMC3443561 22751135

[18] Ren, C. et al. Palmitoylethanolamide-incorporated elastic nano-liposomes for enhanced transdermal delivery and anti-inflammation. *Pharmaceutics.*10.3390/pharmaceutics16070876 (2024). 39065574 10.3390/pharmaceutics16070876 PMC11280357

[19] Petersen, A. L. et al. 17-AAG kills intracellular *Leishmania amazonensis* while reducing inflammatory responses in infected macrophages. *PLoS ONE* **7**, e49496. 10.1371/journal.pone.0049496 (2012). 23152914 10.1371/journal.pone.0049496 PMC3496716

[20] Chen, Z., Ceballos-Francisco, D., Guardiola, F. A., Huang, D. & Esteban, M. Skin wound healing in gilthead seabream (*Sparus aurata* L.) fed diets supplemented with arginine. *Fish Shellfish Immunol.* **104**, 347–358. 10.1016/j.fsi.2020.06.026 (2020). 32544556 10.1016/j.fsi.2020.06.026

[21] Zhou, Y., Liu, G., Huang, H. & Wu, J. Advances and impact of arginine-based materials in wound healing. *J. Mater. Chem. B* **9**, 6738–6750. 10.1039/d1tb00958c (2021). 34346479 10.1039/d1tb00958c

[22] Holowatz, L. A., Thompson, C. S. & Kenney, W. L. L-Arginine supplementation or arginase inhibition augments reflex cutaneous vasodilatation in aged human skin. *J. Physiol.* **574**, 573–581. 10.1113/jphysiol.2006.108993 (2006). 16675494 10.1113/jphysiol.2006.108993 PMC1817757

[23] Calvo, E. et al. Pangenomic changes induced by DHEA in the skin of postmenopausal women. *J. Steroid Biochem. Mol. Biol.* **112**, 186–193. 10.1016/j.jsbmb.2008.10.008 (2008). 19013239 10.1016/j.jsbmb.2008.10.008

[24] Majidian, M., Kolli, H. & Moy, R. L. Management of skin thinning and aging: review of therapies for neocollagenesis; hormones and energy devices. *Int. J. Dermatol.* **60**, 1481–1487. 10.1111/ijd.15541 (2021). 33739464 10.1111/ijd.15541

[25] Ricard-Blum, S. & Ruggiero, F. The collagen superfamily: from the extracellular matrix to the cell membrane. *Parodontol.* **53**, 430–442. 10.1016/j.patbio.2004.12.024 (2005). 10.1016/j.patbio.2004.12.024 16085121

[26] Marucci, G., Buccioni, M., Varlaro, V., Volpini, R. & Amenta, F. The possible role of the nucleoside adenosine in countering skin aging: A review. *BioFactors (Oxford, England)* **48**, 1027–1035. 10.1002/biof.1881 (2022). 35979986 10.1002/biof.1881 PMC9804842

[27] Leonardi, R. & Jackowski, S. Biosynthesis of pantothenic acid and coenzyme A. *EcoSal Plus*10.1128/ecosalplus.3.6.3.4 (2007). 26443589 10.1128/ecosalplus.3.6.3.4 PMC4950986

[28] Ansary, T. M., Hossain, M. R., Kamiya, K., Komine, M. & Ohtsuki, M. Inflammatory molecules associated with ultraviolet radiation-mediated skin aging. *Int. J. Mol. Sci.*10.3390/ijms22083974 (2021). 33921444 10.3390/ijms22083974 PMC8069861

[29] Ressler, S. et al. p16INK4A is a robust in vivo biomarker of cellular aging in human skin. *Aging Cell* **5**, 379–389. 10.1111/j.1474-9726.2006.00231.x (2006). 16911562 10.1111/j.1474-9726.2006.00231.x

[30] Waaijer, M. E. et al. The number of p16INK4a positive cells in human skin reflects biological age. *Aging Cell* **11**, 722–725. 10.1111/j.1474-9726.2012.00837.x (2012). 22612594 10.1111/j.1474-9726.2012.00837.x PMC3539756

[31] Lee, Y. I., Choi, S., Roh, W. S., Lee, J. H. & Kim, T. G. Cellular senescence and inflammaging in the skin microenvironment. *Int. J. Mol. Sci.*10.3390/ijms22083849 (2021). 33917737 10.3390/ijms22083849 PMC8068194

[32] Su, L. et al. 18β-Glycyrrhetinic acid mitigates radiation-induced skin damage via NADPH oxidase/ROS/p38MAPK and NF-κB pathways. *Environ. Toxicol. Pharmacol.* **60**, 82–90. 10.1016/j.etap.2018.04.012 (2018). 29677640 10.1016/j.etap.2018.04.012

[33] Tronino, D. et al. Nanoparticles prolong *N*-palmitoylethanolamide anti-inflammatory and analgesic effects in vivo. *Colloids Surf. B Biointerfaces* **141**, 311–317. 10.1016/j.colsurfb.2016.01.058 (2016). 26866893 10.1016/j.colsurfb.2016.01.058

[34] Singh, A. et al. Topically applied Hsp90 inhibitor 17AAG inhibits UVR-induced cutaneous squamous cell carcinomas. *J. Investig. Dermatol.* **135**, 1098–1107. 10.1038/jid.2014.460 (2015). 25337691 10.1038/jid.2014.460 PMC4366283

[35] Meng, Q., Cooney, M., Yepuri, N. & Cooney, R. N. L-arginine attenuates interleukin-1β (IL-1β) induced nuclear factor kappa-beta (NF-κB) activation in Caco-2 cells. *PLoS ONE* **12**, e0174441. 10.1371/journal.pone.0174441 (2017). 28334039 10.1371/journal.pone.0174441 PMC5363947

[36] Gao, Y. et al. L-arginine attenuates *Streptococcus uberis*-induced inflammation by decreasing miR155 level. *Int. Immunopharmacol.* **130**, 111638. 10.1016/j.intimp.2024.111638 (2024). 38373387 10.1016/j.intimp.2024.111638

[37] Alexaki, V. I. et al. DHEA inhibits acute microglia-mediated inflammation through activation of the TrkA-Akt1/2-CREB-Jmjd3 pathway. *Mol. Psychiatry* **23**, 1410–1420. 10.1038/mp.2017.167 (2018). 28894299 10.1038/mp.2017.167

[38] Cao, J. et al. Dehydroepiandrosterone attenuates LPS-induced inflammatory responses via activation of Nrf2 in RAW264.7 macrophages. *Mol. Immunol.* **131**, 97–111. 10.1016/j.molimm.2020.12.023 (2021). 33461765 10.1016/j.molimm.2020.12.023

[39] Huang, Y. et al. Tryptophan, an important link in regulating the complex network of skin immunology response in atopic dermatitis. *Front. Immunol.* **14**, 1300378. 10.3389/fimmu.2023.1300378 (2023). 38318507 10.3389/fimmu.2023.1300378 PMC10839033

[40] Yu, J. et al. A tryptophan metabolite of the skin microbiota attenuates inflammation in patients with atopic dermatitis through the aryl hydrocarbon receptor. *J. Allergy Clin. Immunol.* **143**, 2108-2119.e2112. 10.1016/j.jaci.2018.11.036 (2019). 30578876 10.1016/j.jaci.2018.11.036

[41] Lohan, S. B. et al. Ultra-small lipid nanoparticles promote the penetration of coenzyme Q10 in skin cells and counteract oxidative stress. *Eur. J. Pharm. Biopharm.* **89**, 201–207. 10.1016/j.ejpb.2014.12.008 (2015). 25500282 10.1016/j.ejpb.2014.12.008

[42] McCart, E. A. et al. Accelerated senescence in skin in a murine model of radiation-induced multi-organ injury. *J. Radiat. Res.* **58**, 636–646. 10.1093/jrr/rrx008 (2017). 28340212 10.1093/jrr/rrx008 PMC5737212

[43] Ma, X. et al. 18β-glycyrrhetinic acid improves high-intensity exercise performance by promoting glucose-dependent energy production and inhibiting oxidative stress in mice. *Phytother. Res. PTR* **35**, 6932–6943. 10.1002/ptr.7310 (2021). 34709693 10.1002/ptr.7310

[44] Veratti, E. et al. 18beta-glycyrrhetinic acid and glabridin prevent oxidative DNA fragmentation in UVB-irradiated human keratinocyte cultures. *Anticancer Res.* **31**, 2209–2215 (2011). 21737643

[45] Liang, M. et al. l-Arginine induces antioxidant response to prevent oxidative stress via stimulation of glutathione synthesis and activation of Nrf2 pathway. *Food Chem. Toxicol.* **115**, 315–328. 10.1016/j.fct.2018.03.029 (2018). 29577948 10.1016/j.fct.2018.03.029

[46] Boo, Y. C. Metabolic basis and clinical evidence for skin lightening effects of thiol compounds. *Antioxidants (Basel, Switzerland).*10.3390/antiox11030503 (2022). 10.3390/antiox11030503 PMC8944565 35326153

[47] Wu, G., Fang, Y. Z., Yang, S., Lupton, J. R. & Turner, N. D. Glutathione metabolism and its implications for health. *J. Nutr.* **134**, 489–492. 10.1093/jn/134.3.489 (2004). 14988435 10.1093/jn/134.3.489

[48] Geria, A. N., Lawson, C. N. & Halder, R. M. Topical retinoids for pigmented skin. *J. Drugs Dermatol. JDD* **10**, 483–489 (2011). 21533293

[49] Gabrielsson, L., Mattsson, S. & Fowler, C. J. Palmitoylethanolamide for the treatment of pain: pharmacokinetics, safety and efficacy. *Br. J. Clin. Pharmacol.* **82**, 932–942. 10.1111/bcp.13020 (2016). 27220803 10.1111/bcp.13020 PMC5094513

[50] Impellizzeri, D. et al. Micronized/ultramicronized palmitoylethanolamide displays superior oral efficacy compared to nonmicronized palmitoylethanolamide in a rat model of inflammatory pain. *J. Neuroinflamm.* **11**, 136. 10.1186/s12974-014-0136-0 (2014). 10.1186/s12974-014-0136-0 PMC4171547 25164769

[51] Shu, P. et al. Efficacy and mechanism of retinyl palmitate against UVB-induced skin photoaging. *Front. Pharmacol.* **14**, 1278838. 10.3389/fphar.2023.1278838 (2023). 37927602 10.3389/fphar.2023.1278838 PMC10622759

[52] Kanehisa, M. & Goto, S. KEGG: kyoto encyclopedia of genes and genomes. *Nucleic Acids Res.* **28**, 27–30. 10.1093/nar/28.1.27 (2000). 10592173 10.1093/nar/28.1.27 PMC102409

[53] Kanehisa, M. Toward understanding the origin and evolution of cellular organisms. *Protein Sci. Publ. Protein Soc.* **28**, 1947–1951. 10.1002/pro.3715 (2019). 10.1002/pro.3715 PMC6798127 31441146

[54] Kanehisa, M., Furumichi, M., Sato, Y., Kawashima, M. & Ishiguro-Watanabe, M. KEGG for taxonomy-based analysis of pathways and genomes. *Nucleic Acids Res.* **51**, D587-d592. 10.1093/nar/gkac963 (2023). 36300620 10.1093/nar/gkac963 PMC9825424

