# Supplementary material for: The anti-aging potential of VA’S new derivatives through metabolomic profiling
Source: Sci Rep. 2025 Nov 27;15:42581. doi: 10.1038/s41598-025-25545-9 (PMC12663416; doi:10.1038/s41598-025-25545-9)
Supplement: Supplementary file 1 — Supplementary Information 1. [file 41598_2025_25545_MOESM1_ESM.docx]

Supplementary Information

**The Anti-Aging Potential of VA’S New Derivatives through Metabolomic Profiling**

*Li Tan^1,#^, Jiang Wu^2,#,*^, Jiewen Wang^1^, Hongkang Zhang^1^, Wei Zhang^2^ , Ang Gao^2^, Zhijian Zhong^4^, Tie-gen Chen^4^, Baoping Zhou^2^, Tengfei Han^2^, Die Zhang^2^, Miao He^3^, Zhenxin Fan^1,*^, Ya Zhou^3,*^*

*^1^ The conservation of endangered wildlife key laboratory of Sichuan province, College of Life Sciences, Sichuan University, Chengdu, China*

*^2^ Shanghai Coachchem Technology, Shanghai, China*

*^3^ Institute of Blood Transfusion, Chinese Academy of Medical Sciences, Chengdu, Sichuan, China*

*^4^ Shanghai Institute of Materia Medica, Chinese Academy of Sciences, Shanghai, China*

^#^ These authors contributed equally

* Correspondence:

Jiang Wu: [johnnywu@coachchem.com](mailto:johnnywu@coachchem.com); Zhenxin Fan: [zxfan@scu.edu.cn](mailto:zxfan@scu.edu.cn); Ya Zhou: zhouya@ibt.pumc.edu.cn


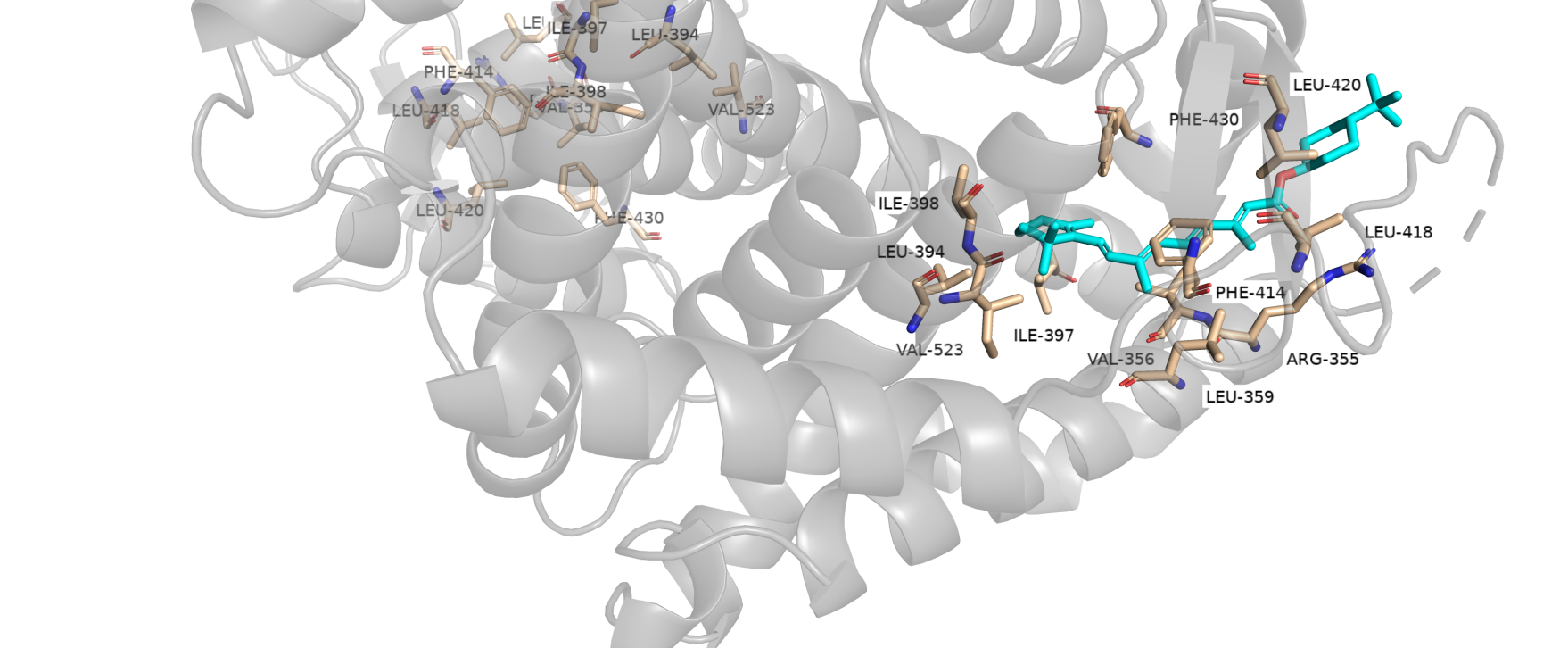


**Fig. S1**. Binding mode of **MVA** with RAR (PDB ID: 6EU9)**.**


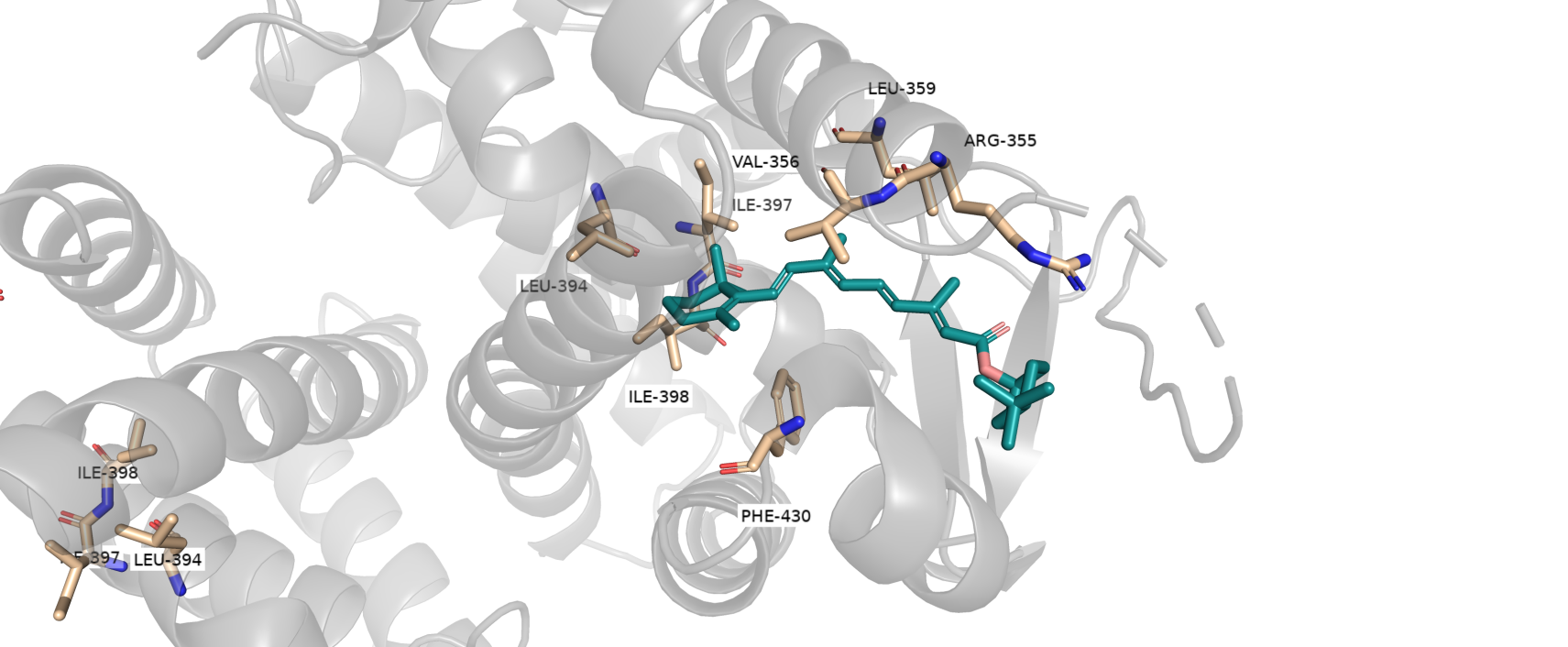


**Fig. S2**. Binding mode of ***iso-* MVA** with RAR (PDB ID: 6EU9)**.**


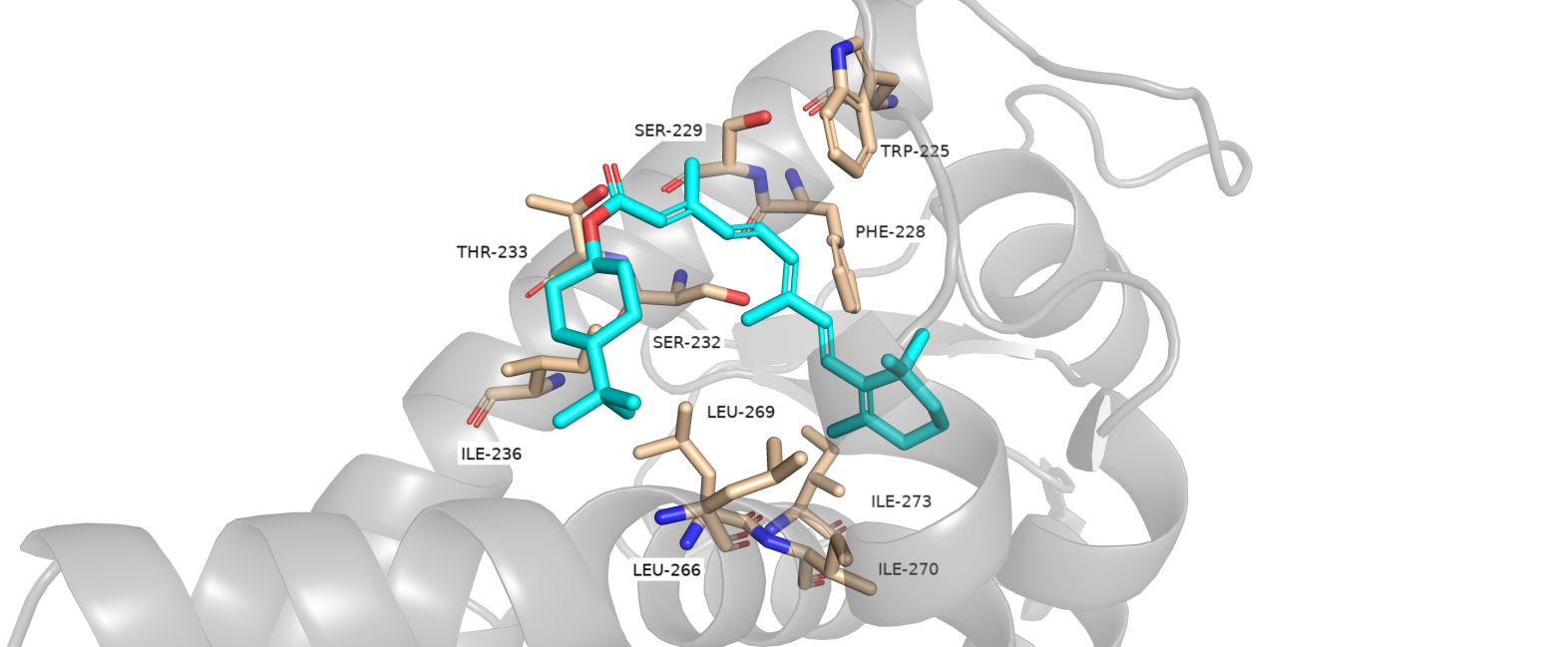


**Fig. S3**. Binding mode of **MVA** with RAR (PDB ID: 3KMZ)**.**


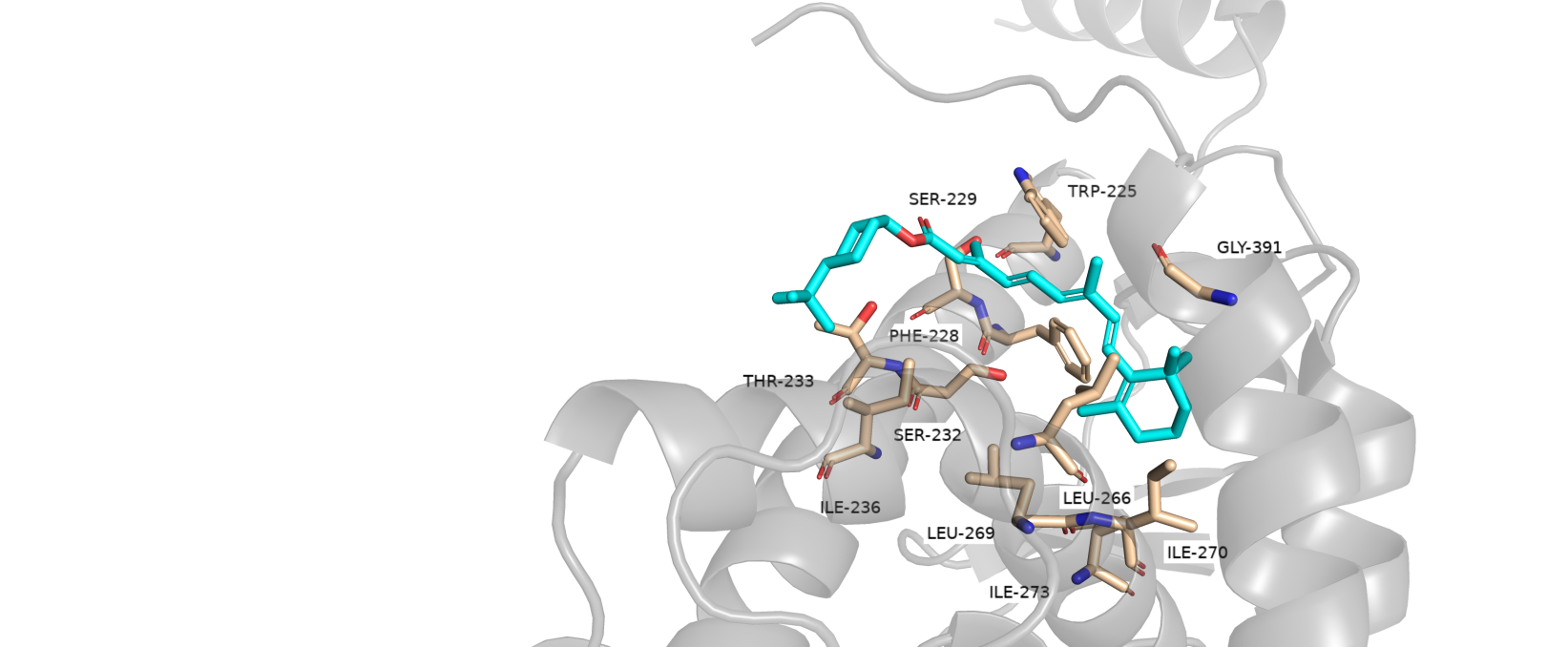


**Fig. S4**. Binding mode of ***iso-*MVA** with RAR (PDB ID: 3KMZ)**.**


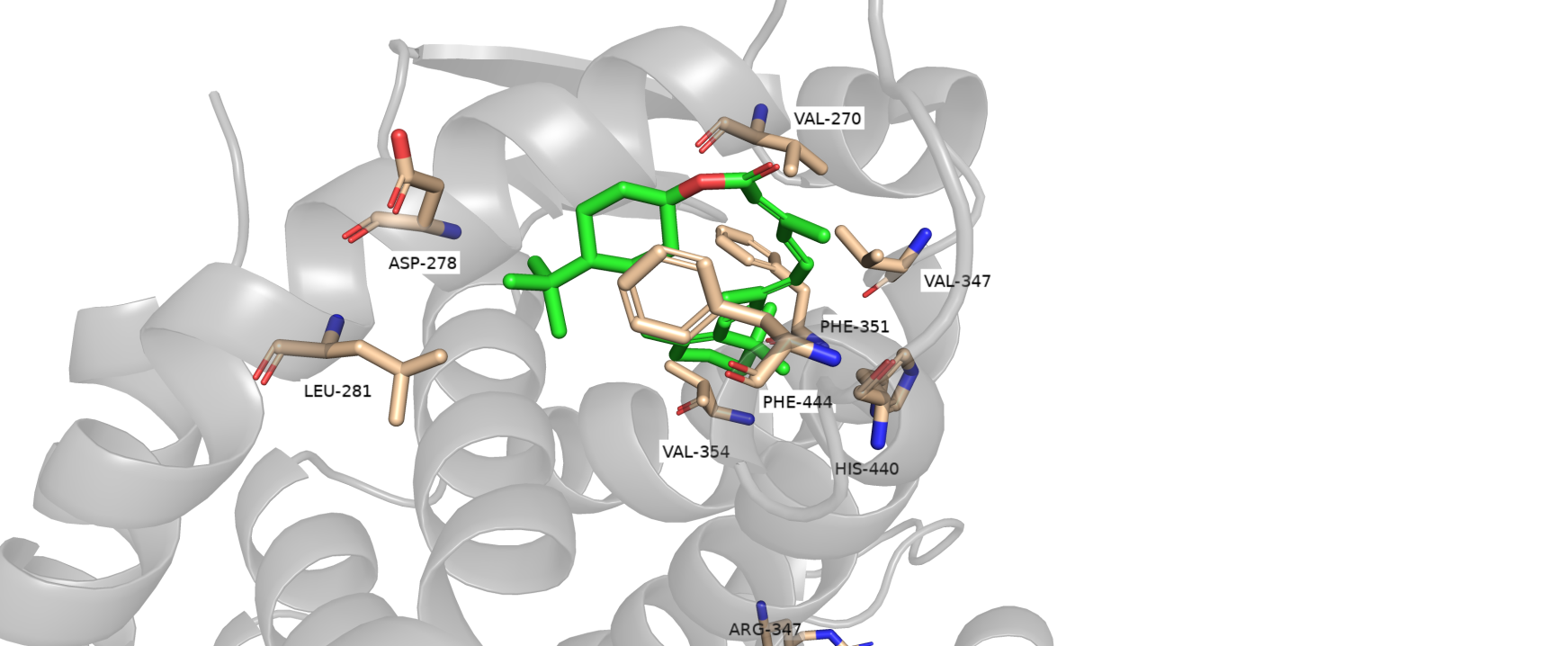


**Fig. S5**. Binding mode of **MVA** with RAR (PDB ID: 3A9E)**.**


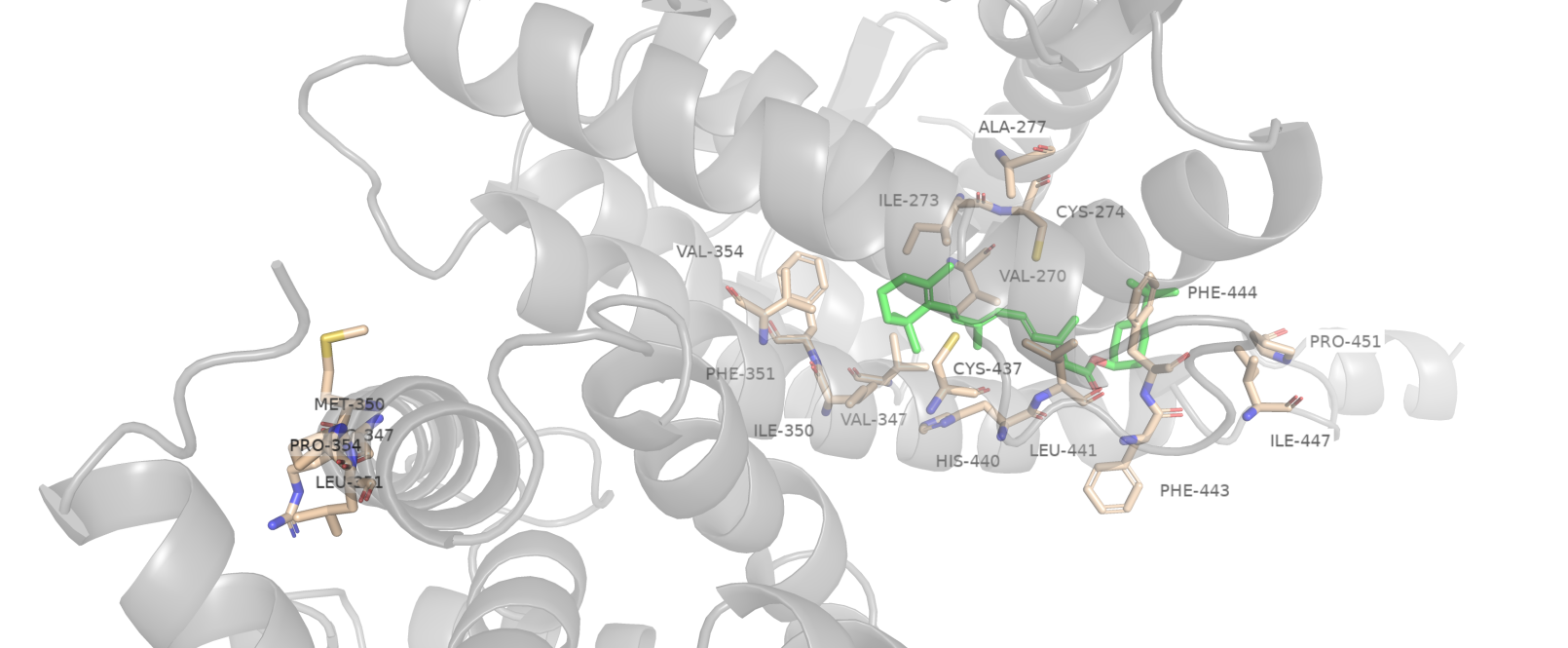


**Fig. S6**. Binding mode of ***iso-*MVA** with RAR (PDB ID: 3A9E)**.**


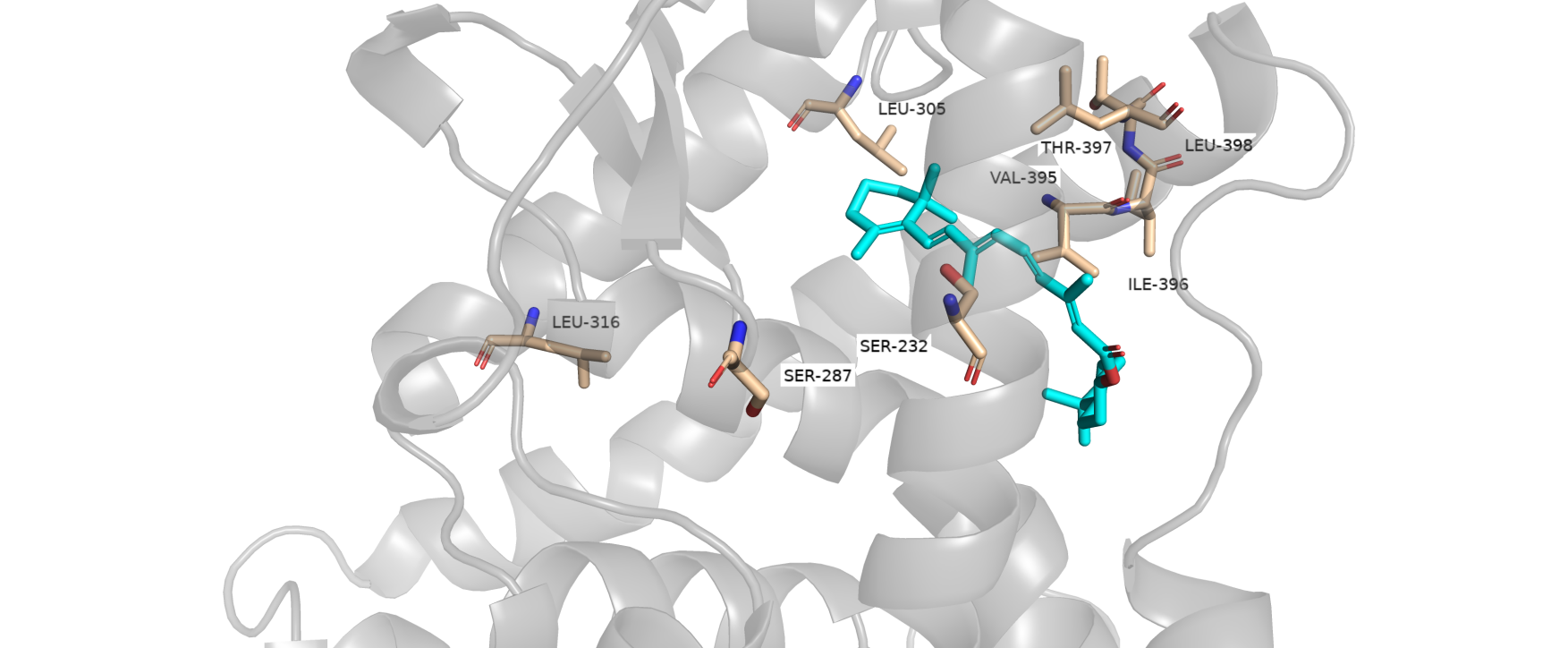


**Fig. S7**. Binding mode of **MVA** with RAR (PDB ID: 1DKF)**.**


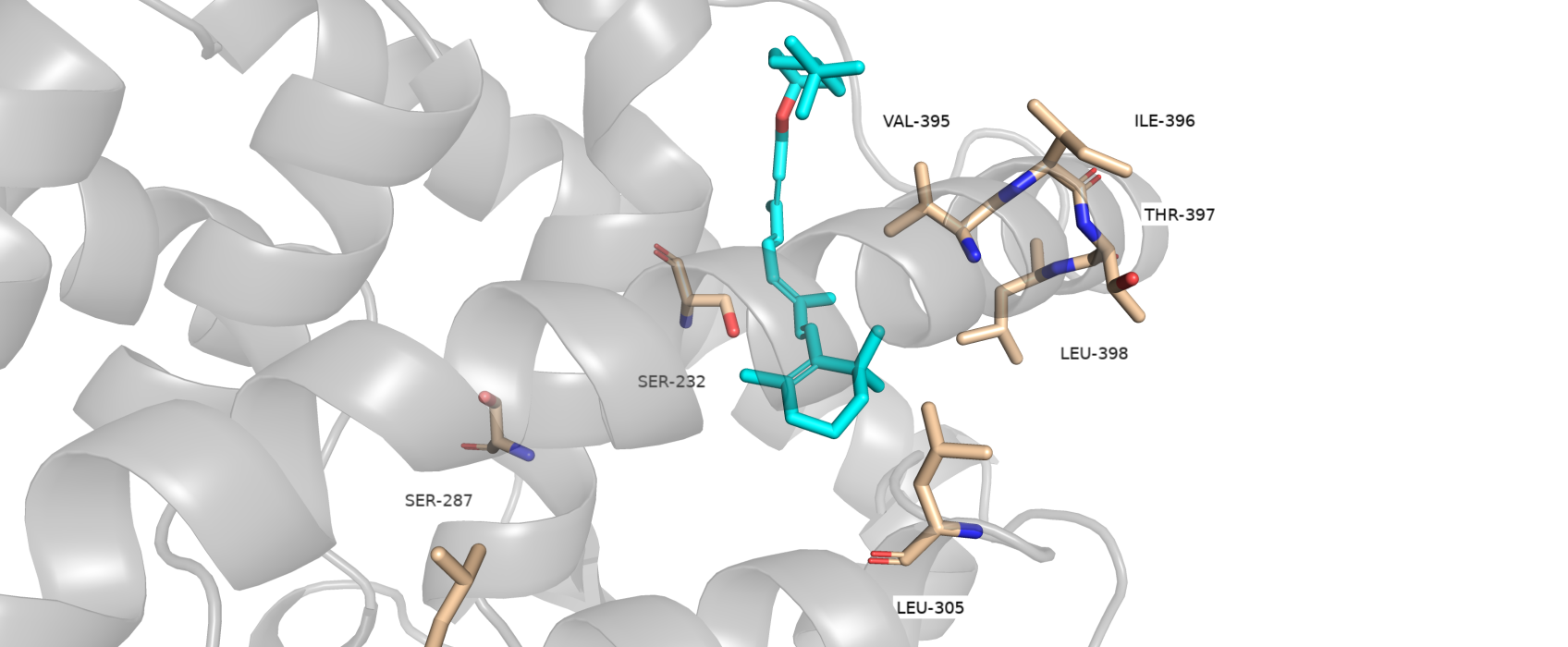


**Fig. S8**. Binding mode of ***iso-*MVA** with RAR (PDB ID: 1DKF)**.**
